# Does Digital Video Advertising Increase Population-Level Reach of Multimedia Campaigns? Evidence From the 2013 Tips From Former Smokers Campaign

**DOI:** 10.2196/jmir.5683

**Published:** 2016-09-14

**Authors:** Kevin C Davis, Paul R Shafer, Robert Rodes, Annice Kim, Heather Hansen, Deesha Patel, Caryn Coln, Diane Beistle

**Affiliations:** ^1^ Center for Health Policy Science and Tobacco Research RTI International Research Triangle Park, NC United States; ^2^ Department of Health Policy and Management Gillings School of Global Public Health University of North Carolina at Chapel Hill Chapel Hill, NC United States; ^3^ Office on Smoking and Health Centers for Disease Control and Prevention Atlanta, GA United States; ^4^ Northrop Grumman Information Systems Atlanta, GA United States

**Keywords:** social marketing, smoking, health campaigns, digital advertising, television advertising

## Abstract

**Background:**

Federal and state public health agencies in the United States are increasingly using digital advertising and social media to promote messages from broader multimedia campaigns. However, little evidence exists on population-level campaign awareness and relative cost efficiencies of digital advertising in the context of a comprehensive public health education campaign.

**Objective:**

Our objective was to compare the impact of increased doses of digital video and television advertising from the 2013 Tips From Former Smokers (Tips) campaign on overall campaign awareness at the population level. We also compared the relative cost efficiencies across these media platforms.

**Methods:**

We used data from a large national online survey of approximately 15,000 US smokers conducted in 2013 immediately after the conclusion of the 2013 Tips campaign. These data were used to compare the effects of variation in media dose of digital video and television advertising on population-level awareness of the Tips campaign. We implemented higher doses of digital video among selected media markets and randomly selected other markets to receive similar higher doses of television ads. Multivariate logistic regressions estimated the odds of overall campaign awareness via digital or television format as a function of higher-dose media in each market area. All statistical tests used the .05 threshold for statistical significance and the .10 level for marginal nonsignificance. We used adjusted advertising costs for the additional doses of digital and television advertising to compare the cost efficiencies of digital and television advertising on the basis of costs per percentage point of population awareness generated.

**Results:**

Higher-dose digital video advertising was associated with 94% increased odds of awareness of any ad online relative to standard-dose markets (*P*<.001). Higher-dose digital advertising was associated with a marginally nonsignificant increase (46%) in overall campaign awareness regardless of media format (*P*=.09). Higher-dose television advertising was associated with 81% increased odds of overall ad awareness regardless of media format (*P*<.001). Increased doses of television advertising were also associated with significantly higher odds of awareness of any ad on television (*P*<.001) and online (*P*=.04). The adjusted cost of each additional percentage point of population-level reach generated by higher doses of advertising was approximately US $440,000 for digital advertising and US $1 million for television advertising.

**Conclusions:**

Television advertising generated relatively higher levels of overall campaign awareness. However, digital video was relatively more cost efficient for generating awareness. These results suggest that digital video may be used as a cost-efficient complement to traditional advertising modes (eg, television), but digital video should not replace television given the relatively smaller audience size of digital video viewers.

## Introduction

In 2012, the US Centers for Disease Control and Prevention (CDC) launched the first federally funded national tobacco education campaign, Tips From Former Smokers (Tips). The campaign aired nationwide on cable television networks in addition to radio, online, print, and out-of-home (eg, billboard) outlets. Tips consisted of evidence-based, graphic, and emotional messages that portrayed the devastating consequences of several smoking- and secondhand smoke-related diseases and conditions, including Buerger’s disease, tracheotomy, and heart attack. All video, radio, online, print, and out-of-home ads from the Tips campaign can be viewed at the Tips website [1]. The campaign was associated with approximately 1.6 million additional quit attempts among US smokers and an estimated 100,000 sustained quits of at least 6 months [2]. In addition, the campaign was found to be highly cost effective based on several accepted thresholds for costs per life year saved [3].

As consumers increasingly use digital devices to view new information and advertising content, public education campaigns have followed suit in using digital media as an advertising platform. In early 2013, CDC launched a second wave of the Tips campaign, which involved similar creative content to that in the 2012 campaign. The 2013 campaign was also supported by a robust digital advertising effort, including online video ads (with the same video content as in the television ads), display ads, mobile ads, and paid search to drive awareness of and traffic to the Tips campaign website. Recently published work has shown that advertising doses on television and digital platforms had significant effects on traffic to the Tips campaign website during the 2013 campaign [4]. Other recent data suggest that exposure to digital advertising during the 2012 Tips campaign was associated with increases in confirmed visits to the Tips campaign website and other cessation-oriented websites for several weeks after exposure [5]. However, gaps remain in understanding digital advertising’s impact on real-world campaign exposure at the population level, practical use as a driver of an overall public health campaign message, and relative cost efficiency. This study extended this work and attempted to address these gaps by directly comparing the effects (and related cost efficiencies) of increased doses of digital video and television advertising on campaign awareness at the population level.

Several studies have examined the use of digital advertising for recruitment to interventions via online ads, social media, and text messaging. These studies found digital advertising to be useful and cost efficient for targeting smokers generally and subpopulations of smokers, such as Latinos, in particular. A 2008 study examined reach and cost effectiveness of a digital campaign to recruit smokers in New Jersey to a cessation treatment program and found that digital advertising yielded costs per enrollee that were competitive with traditional media [6]. In addition, a 2012 study found that digital advertising was cost effective for reaching Spanish-speaking Latino smokers and recruiting them to participate in an online cessation intervention [7].

To date, there is little evidence on the extent to which digital advertising can drive awareness of public education campaigns at the population level. In addition, to our knowledge, no major studies have explored the cost efficiency of digital advertising, relative to traditional broadcast platforms, as part of a comprehensive public health education campaign. However, several studies have demonstrated the potential for digital media to reach tobacco users, as there are already high rates of daily Internet use (82%) and online information seeking about health (80%) among the general adult population in the United States [8]. Google AdWords and search engines were shown to be useful tools for reaching smokeless tobacco users as early as 2005 [9]. In addition, search engine referrals were found to contribute over 70% of traffic to a CDC website on chronic fatigue syndrome over an 18-month period in 2006 and 2007 [10]. As noted above, digital advertising has been shown more recently to be effective at recruiting participants for tobacco cessation interventions [6,11], including among more difficult-to-reach minority subpopulations [7]. Campaign planners must carefully consider the mix of not only the message content, but also the platforms on which those messages will be delivered. In light of constrained budgets for advertising, the relative cost efficiency of advertising platforms is a key element of campaign decision making.

In this study, we compared the impacts of higher doses of digital video and television advertising from the 2013 Tips campaign on overall campaign reach at the population level. This is the first study, to our knowledge, that used a dual-mode design that included higher dosing of digital video and higher dosing of television advertising to identify the independent contributions of each media format to overall campaign awareness. In addition, we used data on adjusted advertising costs for the additional digital video and television advertising doses to compare the cost efficiencies of each format on the basis of costs per percentage point of population awareness generated. These comparisons provide new insights into the role of digital video advertising in driving overall audience exposure in the context of a broad multimedia campaign.

## Methods

### Television Advertising

The 2013 Tips campaign included purchasing advertising on cable television networks, aimed to deliver on average approximately 800 ad gross rating points (GRPs) nationwide. Television ads aired during daytime and primetime hours on television shows frequently watched by the campaign’s target audience of adult smokers. GRPs measure the relative “dose” of advertising delivered to a target audience in a given media market and time period. They are defined as the product of the proportion of an audience that is exposed (ie, audience reach) and the frequency of that exposure (ie, number of times an ad was seen). For example, if a television ad reaches 50% of an audience twice in 1 week, the GRP for this ad during that week is 100 (50 × 2) [12]. In addition to this base national ad buy, we randomly selected 67 designated market areas (DMAs) to receive an additional planned 1600 television GRPs during the 2013 Tips campaign to facilitate a range of analyses on the dose-response impact of additional television advertising.

**Figure 1:**
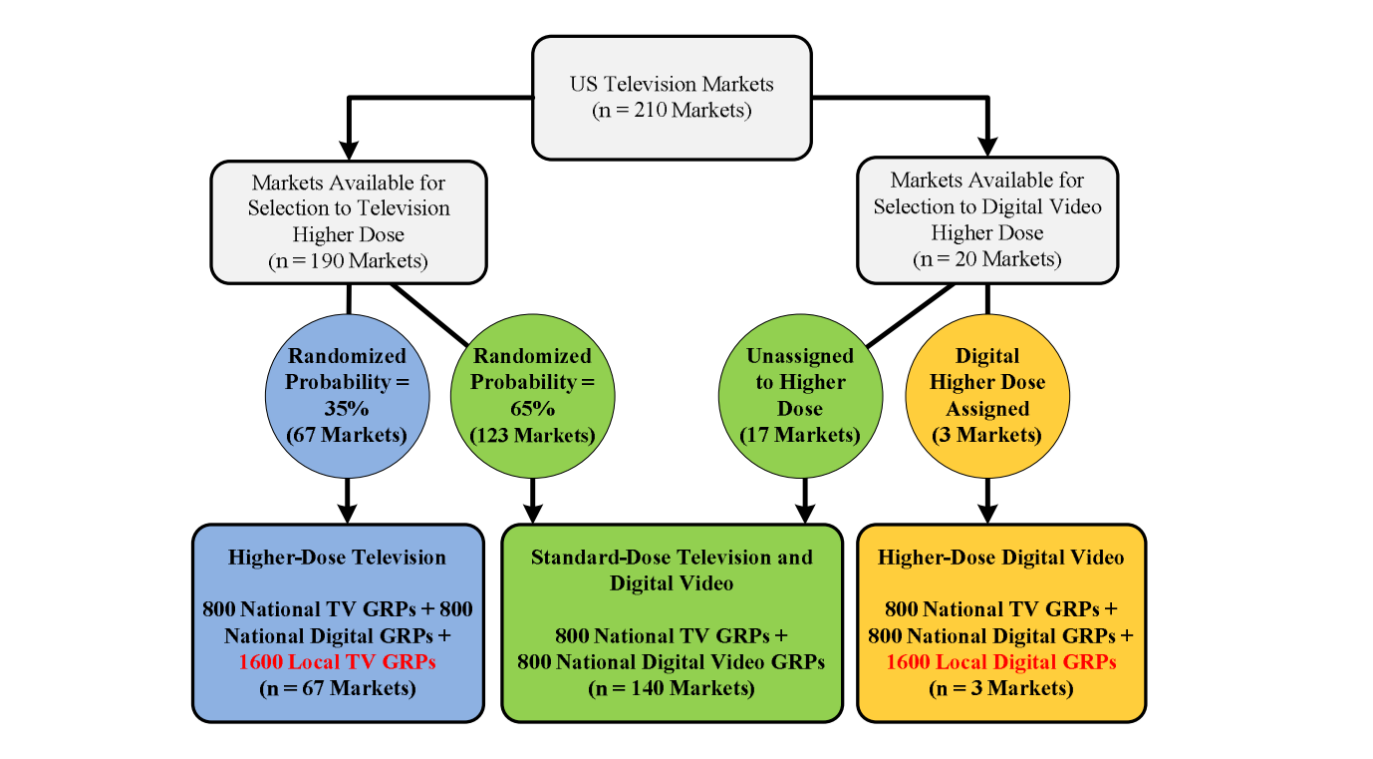
Flow diagram of assignment of designated market areas (DMAs) to higher-dose and standard-dose television and digital video advertising for Tips From Former Smokers 2013 campaign. GRPs: gross rating points.

Figure 1 summarizes the assignment of DMAs to each condition of higher media dosing. We randomly assigned higher-dose television advertising across 190 of the available 210 US DMAs. We excluded the 20 largest US DMAs from this randomization due to the high costs of additional local advertising in these markets. We stratified the remaining 190 DMAs by several characteristics that are associated with smoking, including race/ethnicity, income, and education, and then randomly assigned the DMAs within these strata. The probability of assignment to higher-dose television advertising was set at 35% based on the available budget for local television ad buys, resulting in 67 DMAs assigned to higher-dose television and 123 DMAs assigned to standard-dose television. The television campaign and methods of market-level randomization of the television media dose are discussed in more detail in a recent study of the 2013 Tips television campaign’s impact on cessation-related outcomes among smokers [13].

### Digital Video Advertising

To enable comparisons of costs and ad awareness between television and digital video, we implemented a standard-dose and higher-dose digital video ad buy to mimic the standard-dose and higher-dose television advertising described above. We applied higher-dose digital video advertising within a subset of the remaining 20 largest DMAs that were excluded from the random assignment of higher-dose television described above. The digital video campaign included digital video ads (featuring the same 30-second ads used for the national television campaign) placed on a variety of online advertising networks such as Adotube (Exponential Interactive, Inc., Emeryville, CA, USA) and Tremor (Tremor Video, Inc., New York, NY, USA), as well as several online media networks, including YouTube (YouTube, LLC, San Bruno, CA, USA), Turner networks (Turner Broadcasting System, Inc., Atlanta, GA, USA), and Discovery (Discovery Communications, Inc., Silver Spring, MD, USA). These networks are frequently used by the campaign’s target audience of adult smokers. All digital video ads were clickable and directed viewers to the campaign’s website.

**Figure 2:**
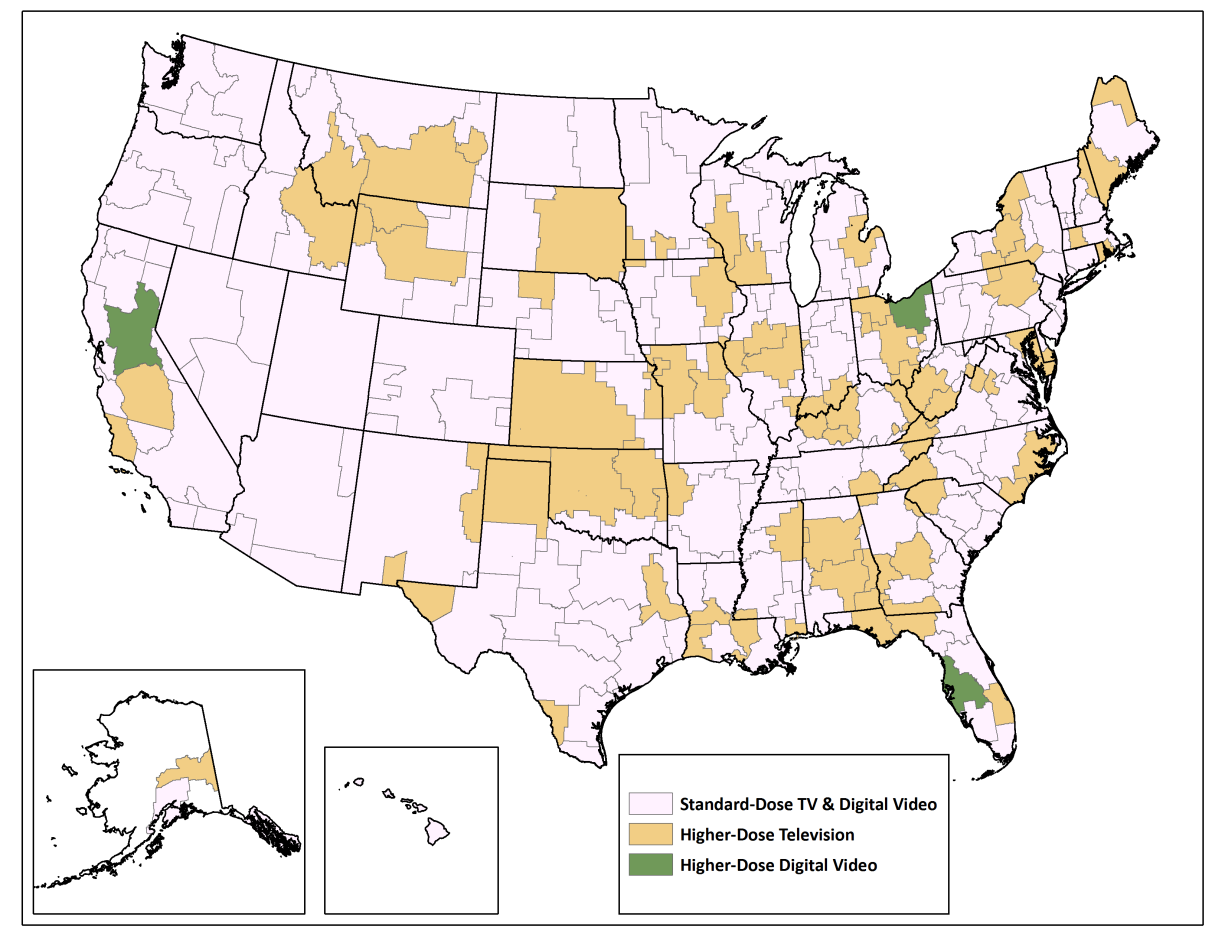
Assignment of media exposure conditions to designated market areas in the United States for the Tips From Former Smokers 2013 campaign.

We chose 3 DMAs—Tampa, Florida; Cleveland, Ohio; and Sacramento, California—to receive a higher dose of digital video advertising of roughly 1600 digital video-equivalent GRPs, mimicking the scale of the higher-dose television ad buy. We geographically targeted digital video ads based on the Internet protocol (IP) address of the users of websites where the ads were placed. In cases where the IP address of the user could not be attributed to a specific media market, that user was not included in the calculation of ad impressions and thus was not counted as a contribution to the digital GRPs. We chose these markets based on the affordability of local digital media buys and to provide some geographic diversity in the group of digital higher-dose markets. Furthermore, the markets selected were very similar in terms of overall target population size, allowing for a nearly equal delivery of digital video GRPs across these markets. The remaining 17 large markets that we excluded from higher-dose television and digital advertising made up the remainder of the standard-dose condition (140 DMAs in total). Figure 2 illustrates the allocation of the 3 media assignment conditions across DMAs in the United States.

### Survey Data

To measure population-level ad exposure rates, we used survey data from a national online survey of cigarette smokers conducted immediately after the conclusion of the 2013 Tips campaign. The survey sample was recruited from the online GfK KnowledgePanel (GfK Custom Research, LLC, Nuremberg, Germany) and included all previously available and newly recruited smokers in this panel. KnowledgePanel is recruited using address-based probability sampling, covering more than 95% of all US households. All panelists have a known probability of selection and cannot volunteer to be a part of the panel. The KnowledgePanel recruitment procedures are described in greater detail elsewhere [2,14,15]. Smokers were defined as adults aged ≥18 years who had smoked at least 100 cigarettes in their lifetime and reported currently smoking either every day or some days at the time of the 2013 Tips launch.

The final analytic dataset included a total of 15,400 current cigarette smokers aged ≥18 years in the United States. By market areas, there were 578 smokers in the 3 higher-dose digital markets and 4632 smokers in the 67 higher-dose television markets. There were 10,190 smokers in total across the remaining 140 markets that received standard-dose television and digital video advertising. We weighted the survey data to be representative of the US adult cigarette smoker population. The weighting procedure used is similar to the weighting methods of the Behavioral Risk Factor Surveillance System, which uses demographic benchmarks from the US Census to yield a weighted survey sample that matches the US Census distributions for age, sex, race/ethnicity, and education [16,17].

### Outcome Variables

The outcome variables in this study include a range of measures of self-reported exposure to the Tips campaign. We measured self-reported exposure to campaign ads using a standard ad recognition protocol [18]. Respondents first viewed 7 of a possible 11 Tips television and digital video ads via video stream within the survey to prompt recall and then immediately completed a battery of questions assessing their exposure to the ad in the past 3 months since the 2013 Tips campaign launch. Those who indicated any awareness of the viewed ad were then asked to report the media format on which they recalled seeing the ad: (1) computer desktop or laptop, (2) mobile device, or (3) television. Respondents could indicate multiple media sources, since all Tips ads were available on television and digital formats. We repeated this process of displaying ads and assessing awareness for each ad, and randomized the display order of the ads. Respondents who were unable to view the ads via the within-survey video stream were shown a storyboard of screenshots from the ad along with text of the ad script. Among all respondents, 86.2% (13,275/15,400) were able to view the video ad streams, while the remaining participants viewed them as screenshots. There was no statistically significant difference in the rate of self-reported ad awareness across these 2 modes of ad viewing.

Using this ad recognition protocol, we created 3 dichotomous indicators of ad awareness within the past 3 months: (1) awareness of any ad via digital formats (computer desktop, laptop, or mobile device), (2) awareness of any ad via television, and (3) awareness of any ad via digital *or* television format. The third measure gauged overall campaign reach. We also created 2 additional dichotomous outcome indicators for awareness of Tips ads exclusively via digital formats and exclusively via television to examine the extent of simultaneous exposure through both formats. Finally, we created an index that measured overall frequency of ad exposure via either digital or television format among individuals who indicated that they had seen at least one Tips ad in the past 3 months. This index was defined as the sum of recall frequency (1=rarely saw ad, 4=saw ad very often) across each of the 7 ads that respondents viewed within the survey. Respondents who saw none of the 7 ads shown received a value of 0, whereas respondents who saw all 7 ads “very often” received a value of 28 for frequency of exposure (total range from 0 to 28).

### Independent Variables

The primary independent variables in our analysis were dichotomous indicators of each media dose area: (1) higher-dose television markets, (2) higher-dose digital markets, and (3) standard-dose television and digital markets. In addition to these variables, we measured a wide range of potential confounders at the individual, state, and media market levels that may have been associated with ad recall. We examined whether these factors varied significantly across the exposure conditions to identify relevant control variables to include in our statistical analysis. These variables included demographic covariates for age, sex, race/ethnicity, education, annual household income, television viewing hours per day, household presence of children aged ≤17 years, household presence of a cigarette smoker, and having a chronic or mental health condition. In addition, we merged external state- and market-level variables with the survey data, including cumulative state per capita tobacco control program funding (1985–2012), state cigarette excise tax (2012), market-level population size, median income (in tens of thousands of dollars), and percentage of the population with a bachelor’s degree. We also measured market-level cigarette smoking prevalence by aggregating recently published county-level data on smoking prevalence [19] to the market level, weighted by county population.

### Advertising Costs

Cost information for the higher doses of television and digital video advertising were provided by the Tips campaign contractor, PlowShare Group (Stamford, CT, USA). These included the total costs for purchasing the higher doses of television and digital video advertising in each geographic area. Because the higher-dose digital markets have a larger audience than higher-dose television markets, average media costs for any platform are higher in the higher-dose digital markets. Therefore, we adjusted costs for the higher dose of digital video advertising for differences in total audience sizes. This was done to create per-market cost estimates that were comparable between the television and digital higher-dose markets. This adjustment was based on the estimated audience size for digital media (ie, total households with broadband Internet access in 2013) across the 67 higher-dose television markets. This enabled the Tips campaign’s advertising agency to calculate the per-market costs for the increased dose of digital advertising as if it had been applied to the 67 higher-dose television markets.

### Statistical Analysis

To assess the impact of additional digital and television advertising on audience exposure across each group of markets, we used logistic regression models to estimate each of the dichotomous awareness variables as a function of higher-dose digital and higher-dose television advertising markets, with standard-dose digital and television markets as the reference category. We used similar linear regression models to estimate the cumulative index of frequency of ad exposure as a function of the media dosing markets. All models included covariates for any of the aforementioned individual, market, or state-level variables that were significantly different across each media dose condition in bivariate analysis. Descriptive analyses of these variables showed that income, presence of a mental health condition, media market population, and media market median income varied significantly across the exposure conditions. Hence, our models included covariates for each of these variables.

Standard errors for each model were clustered at the media market level. We calculated predicted values for the outcome of awareness of any ad in digital or television format to estimate the percentage increases in overall awareness associated with digital and television higher-dose advertising. We used 1-tailed tests as our primary test of significance in the regression models, since our study was a real-world dosing test where all past research, including the 2012 Tips evaluation [2], suggested that there are no reasonable expectations that increased dosing of media would have negative effects on ad awareness [20-25]. All statistical tests used the .05 threshold for statistical significance. Given the smaller sample size of the higher-dose digital exposure condition, we also report marginally nonsignificant results below the .10 level, as these differences may be qualitatively meaningful but have more limited statistical power.

We calculated ad buy costs for each additional percentage point of predicted ad awareness attributable to the higher-dose digital and higher-dose television advertising. We compared costs per additional point of overall ad awareness and frequency of exposure between the increased digital and television advertising doses to assess the marginal benefits of each advertising channel in increasing overall campaign reach at the population level. All analyses were conducted using Stata statistical software, Release 13 (StataCorp LP).

## Results

### Survey Sample Demographics

The unweighted sample was 77.0% (11,864/15,400) non-Hispanic white, 9.1% (1405/15,400) Hispanic, 8.1% (1241/15,400) non-Hispanic black, and 5.8% (890/15,400) non-Hispanic other races/ethnicities. Sample weighting appropriately increases representation of subgroups that are underrepresented in the unweighted data. For example, the weighted race/ethnicity distribution was 67.4% non-Hispanic white, 13.7% Hispanic, 12.1% non-Hispanic black, and 6.8% non-Hispanic other races/ethnicities. Table 1 presents the unweighted and weighted distributions of participants by age, sex, race/ethnicity, and education.

**Table 1:** Sample demographics of smokers, Tips From Former Smokers 2013 evaluation survey (N=15,400).

Characteristics		Unweighted data		Weighted data	
		n (%)	95% CI	%	95% CI
**Age range (years)**					
	18–24	1311 (8.5)	8.1–9.0	13.3	12.4–14.3
	25–34	2872 (18.7)	18.0–19.3	17.5	16.7–18.4
	35–54	6256 (40.6)	39.9–41.4	36.8	35.7–37.9
	55+	4961 (32.2)	31.5–33.0	32.4	31.4–33.5
**Sex**					
	Male	6441 (41.9)	41.1–42.7	48.7	47.5–49.9
	Female	8941 (58.1)	57.3–58.9	51.3	50.1–52.5
**Race/ethnicity**					
	Non-Hispanic white	11,864 (77.0)	76.4–77.7	67.4	66.2–68.7
	Non-Hispanic black	1241 (8.1)	7.6–8.5	12.1	11.3–13.0
	Hispanic	1405 (9.1)	8.7–9.6	13.7	12.7–14.7
	Non-Hispanic other	890 (5.8)	5.4–6.2	6.8	6.2–7.4
**Education**					
	Less than high school	948 (6.2)	5.8–6.6	15.0	13.8–16.3
	High school	3802 (24.7)	24.0–5.4	29.0	27.9–30.0
	Some college	7008 (45.5)	44.7–46.3	30.	29.8–31.6
	College graduate	3642 (23.7)	23.0–24.3	25.4%	24.4–26.3

### Impact of Higher-Dose Digital Video and Television Advertising on Campaign Reach

Table 2 summarizes logistic and linear regression results for the relationships between higher-dose advertising and ad awareness. Higher-dose digital advertising was associated with 94% higher odds of awareness of any ad online relative to standard-dose markets (odds ratio, OR 1.94, *P*<.001). Higher-dose digital advertising was not significantly associated with increased awareness of ads via television (OR 1.33, *P*=.12). Higher-dose digital advertising was associated with a marginally nonsignificant increase in overall campaign awareness regardless of media format (OR 1.46, *P*=.09). Increased doses of television advertising were associated with significantly higher odds of ad awareness via both digital (OR 1.12, *P*=.04) and television formats (OR 1.87, *P*<.001). Higher-dose television advertising was also associated with significantly higher odds of overall ad awareness regardless of media format (OR 1.81, *P*<.001).

Higher-dose digital advertising was associated with significantly higher odds of ad awareness only through online formats (OR 1.93, *P*<.001), while higher-dose television advertising was associated with a higher odds of ad awareness exclusively via television (OR 1.26, *P*<.001). Higher-dose digital advertising was associated with increased frequency of overall campaign exposure via any media channel (b=1.84, *P*=.01), as was higher-dose television advertising (b=2.79, *P*<.001). Based on predicted values from these models, we estimated that higher-dose digital advertising generated an approximate 6.6 percentage point increase in overall campaign awareness, while higher-dose television generated an estimated 8.6 percentage point increase.

**Table 2:** Regression model results for association between 2013 Tips From Former Smokers higher-dose digital and television advertising and ad awareness outcomes.

Media dose indicator (reference: standard-dose digital and television)		Logistic regression adjusted odds ratios					Linear regression coefficients Frequency of exposure on television or online
		Aware of any ad online	Aware of any ad on television	Aware of any ad on television or online	Aware of any ad on television only	Aware of any ad online only	
**Higher-dose digital market**		1.94	1.33	1.46	0.64	1.93	1.84
	95% CI	1.39–2.70	0.82–2.17	0.84–2.52	0.58–0.71	1.40–2.67	0.25–3.42		
	*P* value	<.001	.12	.09	<.001	<.001	.01		
**Higher-dose television market**		1.12	1.87	1.81	1.26	0.38	2.79
	95% CI	0.99–1.28	1.59–2.19	1.55–2.12	1.12–1.41	0.19–0.75	2.09–3.49
	*P* value	.04	<.001	<.001	<.001	.005	<.001

### Digital Video and Television Cost Comparisons

The estimated cost of the additional television ad buy in the higher-dose markets was approximately US $9 million, while the comparable estimated cost of the additional digital ad buy was US $2.9 million (Table 3). Based on these cost estimates, the total estimated cost per additional percentage point of overall campaign reach (awareness of any ad on television or online) generated by the higher dose of digital video advertising was approximately US $440,000. By comparison, the cost of each additional percentage point of population reach generated by the higher dose of television advertising was approximately US $1 million.

**Table 3:** 2013 Tips From Former Smokers campaign costs for higher-dose digital and television advertising.

Media channel for higher dose of advertising	Cost (millions of US$)	Increase in awareness (%)	Estimated cost per percentage point of increased awareness (millions of US$)
Digital	2.9^a^	6.6	0.44
Television	9.0^b^	8.6	1.0

^a^Adjusted higher-dose digital costs.

^b^Actual higher-dose television costs.

## Discussion

### Principal Findings

Our findings suggest that, although digital video advertising may complement the overall reach of a broader multimedia campaign, television remains the strongest driver of overall campaign reach. The additional dosing of digital video advertising generated a significant increase in Tips ad awareness via online channels and also resulted in a marginally nonsignificant increase in overall ad awareness. However, the boost that the additional digital video advertising provided to the overall reach of the campaign was smaller (6.6 additional percentage points of awareness) than the impact of additional television advertising (8.6 additional percentage points of awareness). Awareness of the Tips campaign ads, and the resulting increased awareness of the specific health consequences of smoking, is an important precursor to action in reducing the prevalence and burden of tobacco use. For example, previous evaluation research on the 2012 Tips campaign showed that the campaign was associated with 100,000 new quit attempts among smokers [2], resulting in sizable downstream benefits, including more than 17,000 premature deaths averted and nearly 180,000 quality-adjusted life years saved [3].

In addition, we found that, although higher-dose digital video advertising generated larger increases in awareness via online channels, the higher-dose television advertising was also associated with a significant increase in online awareness. This likely reflects the fact that ad content was identical across digital video and television formats. Hence, primary awareness via television may have resulted in many individuals subsequently searching for the ads online or through social channels, such as Facebook or YouTube, and gaining additional online exposure through those digital activities. For campaign implementation practice, these results suggest that television advertising remains the most effective approach for driving overall campaign exposure at the population level, even when complementary digital video advertising is present. However, digital video advertising may be used to augment the exposure of a comprehensive campaign by reaching digital audiences.

In postestimation analyses, we found that only 2.3% of the sample in higher-dose digital markets reported seeing Tips ads exclusively online. This suggests that almost all persons exposed to Tips ads via digital channels were also exposed to television ads. Given the similarity in content and viewing experience between digital video and television formats, this raises the possibility that accurately differentiating between media platforms may be difficult. Emerging technologies and formats on which video content can be viewed (eg, smart televisions, tablets, and smartphones) may necessitate measures of ad exposure that are not reliant on self-reports due to possible screen or device indifference among viewers. Campaign practitioners should consider using alternative data sources that provide passive measures of exposure to digital advertising as part of their evaluation efforts for these components of the campaign.

Our analysis suggests that the gains generated by higher doses of digital advertising were somewhat more cost efficient than those generated by higher doses of television advertising. Therefore, digital video advertising may be a cost-efficient way to boost the overall reach of a broad multimedia campaign. However, these findings do not imply that digital video is recommended as a predominant mode of advertising. The overall population of those watching digital video on the Internet in the United States is estimated to be 146 million compared with an estimated 285 million who watch traditional television [26]. Hence, the Internet digital video audience is smaller than the traditional television audience and may be younger or otherwise different from the general population, which has implications for the messaging and targeting of public health education campaigns. In addition, digital video advertising is delivered with more precision through targeted buys based on consumer online behaviors and demographic attributes, whereas television advertising is purchased for broader, less-defined audiences. This creates inherent limits to the maximum population reach that is achievable via digital video advertising channels, but it also facilitates more efficient reach among well-defined target audiences.

### Limitations

This study has a few limitations. First, the assignment of higher-dose digital advertising was not randomized. Although we controlled for market-level characteristics that were statistically significant across media dose conditions, there may have been other unobserved and unmeasured factors that were also related to ad exposure. Second, our assessment of the impact of higher-dose digital advertising on recall of campaign ads was based on a relatively smaller sample of smokers in the 3 higher-dose digital markets. Hence, estimates of the effect of higher-dose digital advertising on campaign awareness may be less precise than estimates of the effect of higher-dose television advertising. Third, self-reported exposure to television ads may have been underreported because of our use of a Web-based survey, which potentially underrepresents populations that are not Internet enabled. Awareness of television ads may be underestimated if these populations rely more on television for media exposure. However, as described elsewhere [2,14,15], the KnowledgePanel sample we used provided coverage for non-Internet households by either providing a free laptop and Internet service or other means such as additional honoraria for completing the survey at a location with public Internet access. Fourth, it is possible that there was some imprecision in DMA-based media assignments based on TV media “leakage” between adjacent DMAs or imprecision in the IP-based geographic assignments of digital media [27]. Fifth, the overlap of ad exposures via television and digital formats highlights potential difficulties in measuring the true contributions of digital video advertising as part of a broader multimedia campaign. In the case of the 2013 Tips campaign, the television and digital video ad content were identical. Given the low rates of online-only ad exposure, many individuals who reported seeing Tips ads online may have seen them on television first and subsequently searched for the ads online or discovered the ads after visiting the Tips website (which was also promoted by the television ads) or social media. We do not know the proportion of the smoker population that was exposed to a placed digital ad, as opposed to secondary exposure through searches after initial exposure via television. Additional measures on how digital ads are “discovered” when awareness has been claimed are important to understand the extent to which digital video advertising drives message exposure at the population level.

This is the first study, to our knowledge, to draw direct efficiency comparisons between digital video and television advertising in increasing the overall reach of a multimedia campaign at the population level. Previous studies have primarily focused on the use of digital media for purposes of recruiting smokers to cessation interventions [6,7,9] but have not systematically analyzed the use of digital media for promoting overall campaign reach. This study helps fill this gap with an assessment of digital video advertising from the 2013 Tips campaign and compared its efficiency with that of traditional television advertising.

### Conclusions

In summary, this study provides new evidence on the role of digital video advertising in boosting the overall population-level reach of a multimedia campaign. Our results suggest that the use of digital advertising enhances overall exposure to a large television-based campaign. We also found that digital advertising was more cost efficient than television in driving ad awareness. However, the smaller size of the digital video viewer audience and relatively smaller effect of digital ad exposure relative to television suggests that digital video is best used as a complement to a main television campaign and not as a replacement for television.

In addition, further research is important to better understand the utility of digital video advertising in light of the high concomitancy of digital and television ad exposure. Specifically, new measures are critical to isolate population-level reach attributable solely to digital video advertising. Additional measurement of format-exclusive ads (ie, ads available in only one format or the other) and exogenous measures of ad exposure are also important to assess the extent to which digital video advertising alone can yield significant population-level rates of exposure.

## Abbreviations

CDC: Centers for Disease Control and Prevention
DMA: designated market area
GRP: gross rating point
IP: Internet protocol
OR: odds ratio
Tips: Tips From Former Smokers
